# Can allo-SCT with RIC cure ATLL? Long-term survivors with excellent PS and with heterogenous HTLV-1 proviral load level

**DOI:** 10.1186/1742-4690-8-S1-A33

**Published:** 2011-06-06

**Authors:** Naokuni Uike, Ryuji Tanosaki, Atae Utsunomiya, Ilseung Choi, Jun Okamura

**Affiliations:** 1 Department of Hematology,National Kyushu Cancer Center, Fukuoka, Japan; 2 Stem Cell Transplantation Unit,National Cancer Center Hospital, Tokyo, Japan; 3 Department of Hematology,Imamura Bun-in Hospital, Kagoshima, Japan; 4 Institute for Clinical Research, National Kyushu Cancer Center, Fukuoka, Japan

## Background

Adult T-cell leukemia/lymphoma(ATLL) has so far had a very poor prognosis by chemotherapy. From the long-term observations of our previous clinical trials (NST-1/NST-2; allogeneic hematopoietic stem cell transplantation with reduced-intensity conditioning regimen (RIC) for ATLL patients), we suspect that RIC strategy might have a possible curative power for ATLL patients (pts).

## Objective

We evaluated the safety and feasibility of allogeneic hematopoietic stem cell transplantation with RIC from matched sibling donors (MSD) for ATLL pts using a conditioning regimen consisting of fludarabine and busulfan. Low-dose antithymocyte globulin was added in the 1st study (NST-1), while it was omitted for the 2nd study (NST-2). We present the results of long-term follow-up of the two trials as well as the longitudinal patterns of changes in HTLV-1 proviral load in survivors.

## Patients and methods

Between Apr, 2001 and Feb, 2006, 30 pts ranged from 50 to 67 years of age were enrolled in NST-1(16 pts) and NST-2 (14 pts). After undergoing the conditioning regimen, they received G-CSF-mobilized peripheral blood (PB) stem cells from HLA-matched sibling donors (MSD). Half of the donors were HTLV-1 carriers. The primary end points in both studies were achievement of complete donor chimerism before day 90, and absence of early transplant-related mortality (TRM) before day100. The HTLV-1 proviral load was estimated using PB samples serially after RIC. HTLV-1 proviral DNA was measured by the quantitative PCR amplification of HTLV-1 pX DNA. The detection limit of the HTLV-1 proviral load was 0.5 The overall survival (OS) curve was estimated by the Kaplan–Meier method.

## Results

The results have been already published elsewhere (Okamura, Blood, 2005, Tanosaki, BBMT, 2008, Choi, BMT, 2011) . Ten of the29 evaluable patients have survived for a median of 115 months (range, 85-130 months) after RIC. All of them maintain their good health (Karnofsky PS score ?90%).The majority of survivors have developed the graft-versus host disease (GVHD) (10/10 pts for acute GVHD and 9/10 pts for chronic GVHD). Overall and progression free survival rates at 5 years for the studies were 36% (95% IC, 21 to 51%) and 31% (95% IC, 17 to 45%), respectively. Serial changes in the HTLV-1 proviral load after RIC in the pts are heterogeneous but can be roughly classified into 3 patterns. In the first pattern, seen in 3 pts, the proviral load became undetectable after RIC and continued to remain so. In the second pattern, seen in 3 pts who had received RIC from HTLV-1 negative donors, the proviral load had become undetectable but returned to detectable levels thereafter. Lastly, in the third pattern, seen in 4 pts who had received the grafts from HTLV-1-carrier donors, the proviral load had remained at the carrier level. All the 10 survivors continue to show complete donor chimera during the observation period regardless of the HTLV-1 proviral load level.

## Conclusion

The long-trem follow–up in our study indicates not only that RIC from MSD is a feasible treatment modality for ATL, but also that one third of the pts may be cured with this procedure because all survivors are good for health. As for post-RIC changes in HTLV-1 proviral load in long-term survivors it seems heterogenous, which may be categoryzed into 3 patterns.

